# Risk factors for severe adult-onset asthma: a multi-factor approach

**DOI:** 10.1186/s12890-021-01578-4

**Published:** 2021-07-08

**Authors:** Sanna Toppila-Salmi, Riikka Lemmetyinen, Sebastien Chanoine, Jussi Karjalainen, Juha Pekkanen, Jean Bousquet, Valérie Siroux

**Affiliations:** 1 Haartman Institute, Medicum, University of Helsinki, Haartmaninkatu 3, PO Box 21, 00014 Helsinki, Finland; 2 Skin and Allergy Hospital, Hospital District of Helsinki and Uusimaa, Helsinki University Hospital and University of Helsinki (HUS), Meilahdentie 2, PO Box 160, 00029 Helsinki, Finland; 3 UGA/Inserm U 1209/CNRS UMR 5309 Joint Research Centre Team of Environmental Epidemiology Applied To Reproduction and Respiratory Health, Institute for Advanced Biosciences, Site Santé - Allée Des Alpes, 38700 La Tronche, France; 4 Pôle Pharmacie, CHU Grenoble Alpes, 38000 Grenoble, France; 5 Université Grenoble Alpes, 38000 Grenoble, France; 6 Allergy Centre, Tampere University Hospital, Teiskontie 35, PO Box 2000, 33521 Tampere, Finland; 7 Faculty of Medicine and Health Technology, Tampere University, Tampere University, 33014 Tampere, Finland; 8 Department of Public Health, University of Helsinki, Tukholmankatu 8 B, PO Box 20, 00014 Helsinki, Finland; 9 Environmental Health, National Institute for Health and Welfare, PO Box 95, 70701 Kuopio, Finland; 10 Humboldt-Universität Zu Berlin, and Berlin Institute of Health, Comprehensive Allergy Center, Department of Dermatology and Allergy, Charité, Universitätsmedizin Berlin, Berlin, Germany; 11 University Hospital Montpellier, MACVIA-France, Montpellier, France

**Keywords:** Acetylsalicylic acid, Allergy, Asthma, Epidemiology, Sinusitis

## Abstract

**Background:**

The aim was to identify risk factors for severe adult-onset asthma.

**Methods:**

We used data from a population-based sample (Adult Asthma in Finland) of 1350 patients with adult-onset asthma (age range 31–93 years) from Finnish national registers. Severe asthma was defined as self-reported severe asthma and asthma symptoms causing much harm and regular impairment and ≥ 1 oral corticosteroid course/year or regular oral corticosteroids or waking up in the night due to asthma symptoms/wheezing ≥ a few times/month. Sixteen covariates covering several domains (personal characteristics, education, lifestyle, early-life factors, asthma characteristics and multiple morbidities) were selected based on the literature and were studied in association with severe asthma using logistic regressions.

**Results:**

The study population included 100 (7.4%) individuals with severe asthma. In a univariate analysis, severe asthma was associated with male sex, age, a low education level, no professional training, ever smoking, ≥ 2 siblings, ≥ 1 chronic comorbidity and non-steroidal anti-inflammatory drug (NSAID)-exacerbated respiratory disease (NERD) (p < 0.05), and trends for association (p < 0.2) were observed for severe childhood infection, the presence of chronic rhinosinusitis with nasal polyps, and being the 1st child. The 10 variables (being a 1st child was removed due to multicollinearity) were thus entered in a multivariate regression model, and severe asthma was significantly associated with male sex (OR [95% CI] = 1.96 [1.16–3.30]), ever smoking (1.98 [1.11–3.52]), chronic comorbidities (2.68 [1.35–5.31]), NERD (3.29 [1.75–6.19]), and ≥ 2 siblings (2.51 [1.17–5.41]). There was a dose–response effect of the total sum of these five factors on severe asthma (OR [95% CI] = 2.30 [1.81–2.93] for each one-unit increase in the score).

**Conclusions:**

Male sex, smoking, NERD, comorbidities, and ≥ 2 siblings were independent risk factors for self-reported severe asthma. The effects of these factors seem to be cumulative; each additional risk factor gradually increases the risk of severe asthma.

## Background

The prevalence of asthma has strongly increased over the past decades, and approximately 10% of the population in industrialized countries has had asthma at some point [1]. Asthma is a heterogeneous disease, and among the various asthma characteristics involved in the phenotypic heterogeneity of the disease, both clinical observations and statistical cluster-based approaches identified age at asthma onset as a key differentiating factor [2–5]. Asthma often starts early in life, but asthma can appear in adulthood, and adult-onset asthma has been the focus of less attention. In relation to childhood-onset asthma, adult-onset asthma is associated with more respiratory symptoms, asthma medication use [6] and a poorer prognosis [7]. Adult-onset asthma demonstrates multiple phenotypes, and severe adult-onset asthma is of particular concern and requires further investigation.

Severe asthma is found in approximately 5% to 10% of patients with asthma [8], and a recent Finnish cohort of unselected patients with adult-onset asthma estimated that 5.9% fulfilled the ERS/ATS criteria for severe asthma [9]. Defining severe asthma is difficult, particularly in epidemiological research, since several definitions of severe asthma have been proposed to guide asthma management (GINA, ERS/ATS, WHO), with poor agreement between some of these definitions, and the definitions are often not directly applicable in epidemiological studies [10]. Nevertheless, several risk factors have been proposed for severe asthma in adults, including type 2 inflammation (eosinophilia) [11–13], older age [14], low socio-economic status [15, 16], atopy [16], non-steroidal anti-inflammatory drug (NSAID)-exacerbated respiratory disease (NERD) or NSAID-triggered exacerbation [17], rhinosinusitis associated with nasal polyps [18], sensitization to *Staphylococcus aureus* enterotoxins [19] or to *fungi* [20], smoking or asthma-chronic obstructive pulmonary disease (COPD) overlap [21, 22]. Contradictory results have been observed regarding sex [21, 23]. However, severe adult asthma combines both persistent childhood-onset asthma and adult-onset asthma, two distinct phenotypes, with possibly different risk factors. To our knowledge, few studies have specifically focused on severe adult-onset asthma [14], and except smoking [22], risk factors for severe adult-onset asthma remain poorly characterized.

Early detection of the risk factors contributing to severe adult-onset asthma is important to decrease morbidity and costs. Most previous results have focused on one or a few risk factors, although the phenotypes of asthma are multi-factorial. Hence, as there is still limited knowledge of the putative combination of risk factors in the development of severe adult-onset asthma, this study was carried out to identify risk factors associated with severe asthma in a large population-based case–control study on adult-onset asthma [7, 24]. We hypothesized that factors related to smoking, age, sex, and multimorbidity are positively associated with severe adult-onset asthma and that the risk of severe asthma increases with the number of these risk factors present.

## Methods

### Study design

This is a cross-sectional population-based case–control study of adult-onset asthma in Finland. In 2020, we analysed questionnaire-based data on childhood and adulthood factors. The questionnaire was administered in 1996–97.

### Setting

Population-based sample of asthmatic patients in Finland and their matched controls.

### Study population

**Fig. 1:**
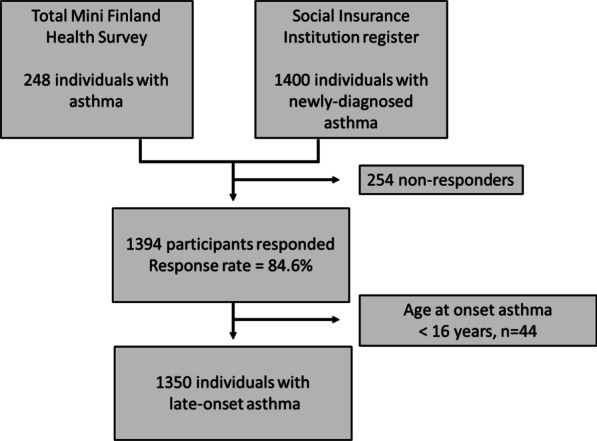
Flowchart of the study population. Asthmatic patients drawn from the Finnish Drug Reimbursement register were over 30 years old. A total of 248 asthmatic patients were included in the longitudinal, population-based Mini Finland Health Survey. A total of 1400 asthmatic patients had recently (within 2 years) been diagnosed with asthma. Asthma was defined as adult onset if the subject responded that the onset of asthma symptoms and/or the age of asthma diagnosis was age 16 years or later

Data from Adult Asthma in Finland, a population-based matched case–control study, were used. The Adult Asthma in Finland study was conducted in 1997 (Fig. 1) as previously described [24]. Data were from 1350 asthma patients older than 30 years of age. Of this population, 182 asthmatic patients were from the longitudinal, population-based Mini Finland Health Survey [25], and 1168 were recently diagnosed asthmatic patients randomly drawn from the Finnish Drug Reimbursement register. The reimbursement right is granted by a certificate that has been made by the patient’s physician and includes background information, clinical exam results, lung function test results and findings and conclusions after an asthma treatment test period of 6 months. All asthmatic patients fulfilled the following criteria for doctor-diagnosed asthma as previously described and validated [26, 27]. Asthmatic patients had self-reported onset of asthma symptoms and/or an asthma diagnosis after 15 years of age. The questionnaire consisted of demographic questions and asthma-specific questions. The proportion of responders in the asthma group was 84.6%. Approval for the study was obtained from the ethical committee at Tampere University Hospital, and written consent was obtained from all subjects.

### Outcomes

The individuals with asthma were asked the year they obtained a diagnosis of asthma from a doctor and the age of the onset of asthma symptoms, a) in childhood, b) at school age, c) over 15 years of age, and d) in adulthood. Among asthmatic patients who fulfilled the selection criteria (detailed above), those who responded that the onset of asthma symptoms and/or the age of asthma diagnosis was 16 years or over were defined as having adult-onset asthma. The mean age (SD, min–max) of doctor-diagnosed asthma was 49.8 (11.8, 16–90) years, and 90.8% reported that their asthma symptoms had started in adulthood (d). Severe asthma (Sev-Q) was defined as self-reported severe asthma and asthma symptoms causing much harm and regular impairment and ≥ 1 oral corticosteroid course/year or regular oral corticosteroid use or waking up in the night due to asthma symptoms/wheezing ≥ a few times/month.

### Covariates

Fifteen covariates were a priori selected based on their potential impact on severe asthma from the data reported in the literature:

- Personal characteristics (3 factors): sex [21, 23, 28], age (< vs. ≥ 50 years) [14], and body mass index (BMI) (< 30 vs. ≥ 30 kg/m^2^) [29, 30]
- Socio-economic characteristics (2 factors) [7]: education level (baccalaureate/secondary vs. primary school) and professional training (completed professional college/university/courses/completed trade school vs. no) [15, 16, 31]
- Lifestyle factors (1 factor) [24]: smoking (never vs. ever) [21, 22, 32]
- Early-life factors (6 factors) [24]: growing up in the countryside/on a farm [33], parental smoking [34], parental asthma and/or allergy [35], severe childhood infections (pneumonia before or during school age or hospitalization due to infection at ≤ 3 years of age) [36], the number of siblings (< 2 vs. ≥ 2) [24], and birth order (1st vs. other) [24, 37, 38]
- Asthma characteristics (1 factor) [39]: non-steroidal anti-inflammatory drug (NSAID) – exacerbated respiratory disease (NERD) [17]

Chronic comorbidities (3 factors) [24]: ≥1 other allergic disease ever [e.g., allergic rhinitis (AR)/allergic conjunctivitis (AC)/atopic dermatitis (AD)] [16, 24], nasal polyps (NPs) [18], and ≥1 other chronic disease(s) [7, 21, 32]. Information on other chronic diseases was obtained by the following questions” Do you have any of the following doctor-diagnosed conditions? Hypertension, coronary artery disease, rheumatoid arthritis, psychiatric disorder, diabetes, ocular hypertension, back disease, arthritis. Has your doctor diagnosed you with any of the following lung diseases? chronic bronchitis, emphysema, bronchiectasis, allergic bronchopulmonary aspergillosis (empyema was only included). Do you have any other doctor-diagnosed chronic diseases? (bronchitis/bronchiectasis was excluded).

### Statistical analysis

The associations between each risk factor and severe asthma were estimated using chi square (dichotomous) and t-tests (continuous) and univariate logistic regressions. Odds ratios (ORs) with 95% confidence intervals are reported. Risk factors associated with severe asthma with a p value below 0.2 were included in a multiple logistic regression model. Risk factors that were statistically significantly associated with severe asthma (p < 0.05) in the multiple logistic regression were combined in a risk score defined as the sum of the risk factors. The association between the risk score and severe asthma was assessed by a logistic regression model. A sensitivity analysis was conducted to address the robustness of the association with the definition of severe asthma, particularly using a medication-based definition of severe asthma (Sev-OCS) with severe asthma defined by the report of oral corticosteroid in regular use or ≥ 2 courses/year due to asthma. Sensitivity analyses were also performed by a more stringent definition of severe asthma by including only those subjects who reported regular use of inhaled corticosteroid (ICS) medication (Sev-Q + ICS, Sev-OCS + ICS). Statistics were performed with SPSS Base 24 Statistical Software Package (SPSS, Chicago, IL, USA).

## Results

### Population description

The study flowchart is shown in Fig. 1. The total number of adult-onset asthma cases with available data was 1350. The mean age (SD, min–max) was 54.4 (12.2, 31–93) years. The proportion of females was 62.1%, and the proportion of subjects reporting at least a secondary school level of education was 36.1%. A hundred subjects (7.4%) reported severe asthma. Severe asthma was associated with an increased number of work impairment days due to respiratory symptoms (1.006 [1.003–1.009] for each one-day increase in the number of work impairment days, p < 0.001). Nineteen (19.0%) severe asthmatic patients and 109 (8.7%) non-severe asthmatic patients reported over 20 work impairment days/year due to respiratory symptoms (p = 0.002).

### Risk factors for severe adult-onset asthma

**Fig. 2:**
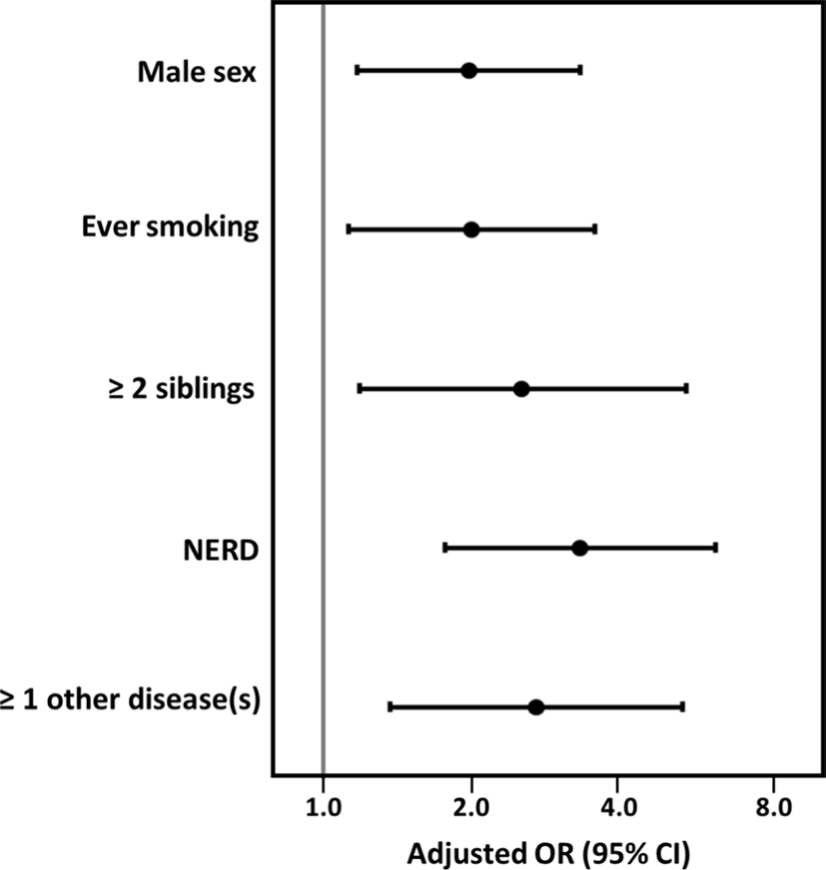
Forest plot summarizing the associations between risk factors and severe adult-onset asthma by using a question-based definition of severe asthma (Sev-Q). Adjusted ORs and 95% CIs of severe adult-onset asthma are presented for sex, smoking status, the number of siblings, the presence of patient-reported NSAID-exacerbated respiratory disease (NERD), and other chronic diseases. Models were adjusted for all these risk factors and age, education, training, severe childhood infection(s)^1^ and the presence of chronic rhinosinusitis with nasal polyps. In this multivariable model, the number of severe asthmatic patients was 79, and the number of non-severe asthmatic patients was 1094. ^1^pneumonia before or during school age and/or hospitalization due to infection at ≤ 3 years of age. Education level = baccalaureate/secondary versus primary school; professional training = completed professional college/university/courses/completed trade school versus no. OR = odds ratio. CI = confidence interval. Severe asthma (Sev-Q) was defined as self-reported severe asthma and asthma symptoms causing much harm and regular impairment and ≥ 1 oral corticosteroid course/year or regular oral corticosteroids and/or waking up in the night due to asthma symptoms/wheezing ≥ a few times/month

The description of self-reported demographic factors in the severe and non-severe asthma groups is shown in Table 1. In unadjusted analysis, severe adult-onset asthma was statistically significantly associated with male sex, older age, low education, no professional training, ever smoking, ≥ 2 siblings, ≥ 1 chronic comorbidity and non-steroidal anti-inflammatory drug (NSAID)-exacerbated respiratory disease (NERD) (p < 0.05, Table 2, Model 1). In addition, there was a trend for an association (p < 0.2) with severe childhood infection, NPs, and being the 1st child (Table 2, Model 1). The variable being the 1^st^ child was strongly associated with ≥ 2 siblings and therefore was not considered in the multivariate analysis due to multicollinearity (Table 2). Thus, the 10 variables were entered in a multivariate regression model, and severe adult-onset asthma (Sev-Q) was significantly associated with male sex (OR [CI95%] = 1.96 [1.16–3.30]), ever smoking (1.98 [1.11–3.52]), chronic comorbidities (2.68 [1.35–5.31]), NERD (3.29 [1.75–6.19]), and ≥ 2 siblings (2.51 [1.17–5.41]) (p < 0.05, Table 2, Model 2 and Fig. 2). When performing analyses by a more stringent definition of severe asthma by including only 88/110 subjects with regular use of inhaled corticosteroids (Sev-Q + ICS), the results remained similar. The 10 variables were entered in a multivariate regression model, and severe adult-onset asthma (Sev-Q + ICS) was significantly associated with male sex (OR [CI95%] = 1.78 [1.03–3.04]), ever smoking (1.93 [1.07–3.49]), chronic comorbidities (2.71 [1.33–5.56]), NERD (3.49 [1.85–6.60]), and ≥ 2 siblings (2.25 [1.04–4.87]) (p < 0.05).

**Table 1:** Association between self-reported demographic factors and severe and non-severe asthma groups

	Non-severe asthma N = 1250	Severe asthma N = 100	P
*Personal characteristics*			
Female sex, n (%)	793 (63.4)	45 (45.0)	** < .001**
Age, years, mean(SD)	54.0 (12.2)	58.7 (11.6)	**.001**
BMI^1^, mean (SD)	26.9 (4.7)	27.1 (5.5)	.94
*Socio-economic characteristics*			
Baccalaureate/secondary school, n (%)	754 (61.8)	71 (76.3)	**.005**
Professional training, n (%)	296 (26.0)	35 (40.7)	**.004**
*Lifestyle factors*			
Ever smokers, n (%)	720 (57.6)	74 (74.0)	**.001**
*Early-life factors*			
1^st^ child, n (%)	363 (29.0)	22 (22.0)	.14
≥ 2 siblings, n (%)	927 (75.9)	84 (89.4)	**.003**
Growing up in the countryside/on a farm, n (%)	937 (75.9)	77 (80.2)	.39
Severe childhood infections^2^, n (%)	217 (18.0)	11 (11.7)	.13
Parental smoking, n (%)	648 (51.8)	53 (53.0)	.84
Parental asthma and/or allergy, n (%)	437 (36.1)	29 (29.9)	.23
*Asthma characteristics*			
NERD, n (%)	124 (9.9)	19 (19.0)	**.007**
*Multimorbidity*			
CRSwNP, n (%)	145 (11.6)	7 (7.0)	.19
≥ 1 other disease^3^, n (%)	843 (67.4)	86 (86.0)	** < .001**
≥ 1 other allergic disease (AR/AC/AD), n (%)	844 (67.5)	63 (63.0)	.38

Bold values denote statistical significance at the *p* < 0.05 level

NERD = patient-reported NSAID-exacerbated respiratory disease; CRSwNP = chronic rhinosinusitis with nasal polyps; AR = allergic rhinitis; AC = allergic rhinoconjunctivitis, AD = atopic dermatitis. P values by Chi square test (dichotomous) or t-test (continuous variables). P values less than 0.05 were considered significant. ^1^BMI data were missing from 6 (6.0%) severe asthmatic patients and 29 (2.3%) non-severe asthmatic patients. ^2^pneumonia before or during school age and/or hospitalization due to infection at ≤ 3 years of age. ^3^Hypertension (n = 298), coronary artery disease (n = 120), rheumatoid arthritis (n = 60), psychiatric disorder (n = 86), diabetes (n = 54), glaucoma (n = 49), back disease (n = 367), arthritis (n = 244), empyema (n = 106), other chronic disease(s) except chronic bronchitis/bronchiectasis (n = 449). Education level = baccalaureate/secondary versus primary school; professional training = completed professional college/university/courses/trade school versus no professional training. Severe asthma (Sev-Q) was defined as self-reported severe asthma and asthma symptoms causing much harm and regular impairment and ≥ 1 oral corticosteroid course/year or regular oral corticosteroids and/or waking up in the night due to asthma symptoms/wheezing ≥ a few times/month. Missing values were included and regarded as “no”

**Table 2:** Association of risk factors with severe adult-onset asthma by using a question-based definition of severe asthma (Sev-Q)

	Model 1 Univariate regression model		Model 2 Multiple regression model n = 1173	
	OR_1_ (95% CI)	p_1_	OR_2_ (95% CI)	p_2_
*Male sex*				
No Yes	1 2.12 (1.41–3.20)	** < .001**	1 1.96 (1.16–3.30)	**.011**
*Age*^1^				
	1.033 (1.02–1.05)	** < .001**	1.002 (0.98–1.03)	.88
*BMI*^1^				
	1.010 (0.97–1.06)	.66	Not entered	
*Baccalaureate/secondary school*				
No Yes	1 0.50 (0.31–0.82)	**.006**	1 0.88 (0.48–1.65)	.70
*Professional training*				
No Yes	1 0.51 (0.33–0.80)	**.003**	1 0.62 (0.36–1.05)	.074
*Ever smokers*				
No Yes	1 2.10 (1.32–3.32)	**.002**	1 1.98 (1.11–3.52)	**.020**
*1st child*				
No Yes	1 0.69 (0.42–1.12)	**.14**	Not entered^2^	
* ≥ 2 siblings*				
No Yes	1 2.66 (1.37–5.20)	**.004**	1 2.51 (1.17–5.41)	**.018**
*Growing up in the countryside/on a farm*				
No Yes	1 1.29 (0.77–2.17)	.34	Not entered	
*Severe childhood infections*^*2*^				
No Yes	1 0.60 (0.32–1.15)	**.13**	1 0.55 (0.26–1.14)	.11
*Parental smoking*				
No Yes	1 1.05 (0.70–1.58)	.82	Not entered	
*Parental asthma and/or allergy*				
No Yes	1 0.76 (0.48–1.19)	.22	Not entered	
*NERD*				
No Yes	1 2.13 (1.25–3.63)	**.005**	1 3.29 (1.75–6.19)	** < .001**
*CRSwNP*				
No Yes	1 0.57 (0.26–1.26)	**.17**	1 0.54 (0.22–1.30)	.17
* ≥ 1 other disease *^*3*^				
No Yes	1 2.97 (1.67–5.28)	** < .001**	1 2.68 (1.35–5.31)	**.005**
* ≥ 1 other allergic disease (AR/AC/AD)*				
No Yes	1 0.82 (0.54–1.25)	.36	Not entered	

Bold values denote statistical significance at the p < 0.05 level

Model 1 = univariate analysis. Model 2 = multivariable analysis by the eleven variables that were associated at the p < 0.2 level in Model 1. NERD = patient-reported NSAID-exacerbated respiratory disease; CRSwNP = chronic rhinosinusitis with nasal polyps; AR = allergic rhinitis; AC = allergic rhinoconjunctivitis, AD = atopic dermatitis. ^1^continuous variables. ^2^The variables “1st child” and “ ≥ 2 siblings” correlated (p < 0.01, r = − 0.42, by Pearson’s correlation test); hence, to avoid multicollinearity, only the statistically significant variable “ ≥ 2 siblings” was added into the multivariable model. ^2^pneumonia before or during school age and/or hospitalization due to infection at ≤ 3 years of age.  ^3^Hypertension (n = 298), coronary artery disease (n = 120), rheumatoid arthritis (n = 60), psychiatric disorder (n = 86), diabetes (n = 54), glaucoma (n = 49), back disease (n = 367), arthritis (n = 244), empyema (n = 106), other chronic disease(s) except chronic bronchitis/bronchiectasis (n = 449). Education level = baccalaureate/secondary versus primary school; professional training = completed professional college/university/courses/trade school versus no professional training. OR = odds ratio. CI = confidence interval. Severe asthma (Sev-Q) was defined as self-reported severe asthma and asthma symptoms causing much harm and regular impairment and ≥ 1 oral corticosteroid course/year or regular oral corticosteroids and/or waking up in the night due to asthma symptoms/wheezing ≥ a few times/month

When counting the total sum of the five risk factors, 27 (2.0%), 188 (13.9%), 433 (32.1%), 398 (29.5%), 260 (19.3%) and 9 (0.7%) had 0, 1, 2, 3, 4, and 5 risk factors, respectively. The risk for severe adult-onset asthma significantly and gradually increased with the sum of the five risk factors (OR (95% CI) = 2.30 [1.81–2.93] for each additional unit, p < 0.001). A significant dose–response effect was also detected by using this sum variable (Table 3) and, when a more stringent definition of severe asthma (Sev-Q + ICS) was added to the model (Table 3).

**Table 3:** Association of the sum of the risk factors (categorized as 0–1, 2–3, and 4–5 risk factors) with severe adult-onset asthma by using a question-based definition of severe asthma (Sev-Q)

	N_1_ total (n with severe asthma)	OR_1_ (95% CI)	p_1_	N_2_ total (n with severe asthma)	OR_2_ (95% CI)	p_2_
0–1	215 (3)	1		215 (3)	1	
2–3	831 (47)	4.24 (1.31–13.75)	**.016**	829 (45)	4.06 (1.25–13.18)	**.020**
4–5	269 (44)	13.82 (4.23–45.18)	** < .001**	262 (37)	11.62 (3.53–38.25)	** < .001**

Bold values denote statistical significance at the *p* < 0.05 level

Risk factors were male sex, ≥ 2 siblings, ever smoking, NERD, and ≥ 1 other disease. When counting the total sum of the five risk factors, 27 (2.0%), 188 (13.9%), 433 (32.1%), 398 (29.5%), 260 (19.3%) and 9 (0.7%) had 0, 1, 2, 3, 4, and 5 risk factors, respectively. The risk for severe asthma (Sev-Q) significantly and gradually increased with the sum of the five risk factors (OR (95% CI) = 2.30 [1.81–2.93] for each additional unit, p < 0.001). NERD = patient-reported NSAID-exacerbated respiratory disease. OR = odds ratio. CI = confidence interval. Severe asthma (Sev-Q) was defined as self-reported severe asthma and asthma symptoms causing much harm and regular impairment and ≥ 1 oral corticosteroid course/year or regular oral corticosteroids and/or waking up in the night due to asthma symptoms/wheezing ≥ a few times/month. ^1^Complete patients in whom the variable data were available. ^2^More stringent definition of severe asthma by including only those subjects with regular use of inhaled corticosteroid (Sev-Q + ICS)

### Sensitivity analysis

The total number of severe asthmatic patients by using an oral corticosteroid-based definition was 226 (16.7%). Forty-one (41.0%) of the questionnaire-based (Sev-Q) severe asthmatic patients also had oral corticosteroid-based (Sev-OCS) severe asthma (p < 0.001). Thirty-seven (16.4%) severe asthmatic patients (Sev-OCS) and 91 (8.1%) non-severe asthmatic patients reported over 20 work impairment days/year due to respiratory symptoms (p = 0.001).

In univariate models, Sev-OCS was associated with ever smoking, being the first child, growing up in the countryside/on a farm, ≥ 1 chronic comorbidity at the p < 0.05 level and with a trend (e.g., at the p < 0.2 level) for high BMI, parental smoking, severe childhood infection, and NERD (Table 4, Model 1). Age (p = 0.20) and sex (p = 0.21) were entered in the multivariate model for comparability with the main analysis. When entering these 10 variables in a multivariate logistic regression model, severe adult-onset asthma (Sev-OCS) was significantly associated with ever smoking, growing up in the countryside/on a farm, NERD, and ≥ 1 other chronic disease (p < 0.047, Table 4, Model 2). When performing analyses by a more stringent definition of severe asthma by including only 203/226 subjects with regular use of inhaled corticosteroids (Sev-OCS + ICS), the results remained relatively similar. The 10 variables were entered in a multivariate regression model, and severe adult-onset asthma (Sev-Q + ICS) was significantly associated with ever smoking (1.64 [1.14–2.36]), ≥ 1 other chronic disease (1.57 [1.06–2.34]) and NERD (1.73 [1.07–2.81]), (p < 0.05), yet growing up in the countryside/on a farm was not significant (0.71 [0.49–1.04], p = 0.08).

**Table 4:** Association of risk factors with severe adult-onset asthma by using a medication-based definition of severe asthma (Sev-OCS). Multivariate model

	Model 1 Univariate regression model		Model 2 Multiple regression model n = 1173	
	OR_1_ (95% CI)	p_1_	OR_2_ (95% CI)	p_2_
*Male sex*				
No Yes	1 1.20 (0.90–1.61)	**.21**	1 1.03 (0.73–1.46)	.87
*Age*^1^	1.008 (1.00–1.02)	**.20**	1.012 (1.00–1.03)	.092
*BMI*^1^	1.03 (1.00–1.06)	**.059**	1.31 (0.92–1.87)	.13
*Baccalaureate/secondary school*				
No Yes	1 0.86 (0.63–1.17)	.33	Not entered	
*Professional training*				
No Yes	1 0.81 (0.58–1.12)	.20	Not entered	
*Ever smokers*				
No Yes	1 1.70 (1.25–2.31)	**.001**	1 1.69 (1.19–2.40)	**.003**
*1st child*				
No Yes	1 1.37 (1.01–1.86)	**.043**	1 1.34 (0.96–1.86)	.088
* ≥ 2 siblings*				
No Yes	1 0.92 (0.65–1.29)	.62	Not entered	
*Growing up in the countryside/on a farm*				
No Yes	1 0.72 (0.52–0.99)	**.041**	1 0.69 (0.48–0.99)	**.046**
*Severe childhood infections*^*2*^				
No Yes	1 1.28 (0.89–1.84)	**.19**	1 1.03 (0.69–1.54)	.88
*Parental smoking*				
No Yes	1 1.23 (0.92–1.64)	**.16**	1 1.24 (0.90–1.69)	.19
*Parental asthma and/or allergy*				
No Yes	1 1.02 (0.75–1.37)	.92	Not entered	
*NERD*				
No Yes	1 1.37 (0.89–2.11)	**.15**	1 1.68 (1.06–2.69)	**.029**
*CRSwNP*				
No Yes	1 0.98 (0.62–1.54)	.92	Not entered	
* ≥ 1 other disease*^*3*^				
No Yes	1 1.73 (1.24–2.43)	**.001**	1 1.49 (1.03–2.18)	**.037**
* ≥ 1 other allergic disease (AR/AC/AD)*				
No Yes	1 1.14 (0.83–1.55)	.42	Not entered	

Bold values denote statistical significance at the p < 0.05 level

Model 1 = Univariate analysis. Model 2 = Multivariable analysis by the twelve variables that were associated at the p < 0.2 level in Model 1). In addition, we wanted to force age (p = 0.20) and sex (p = 0.021) in model 2 to obtain a more comparable model to the first model. Hence, a total of 10 variables were entered in a multivariable model. NERD = patient-reported NSAID-exacerbated respiratory disease; CRSwNP = chronic rhinosinusitis with nasal polyps; AR = allergic rhinitis; AC = allergic rhinoconjunctivitis, AD = atopic dermatitis. ^1^continuous variables. ^2^pneumonia before or during school age and/or hospitalization due to infection at ≤ 3 years of age. ^3^Hypertension (n = 298), coronary artery disease (n = 120), rheumatoid arthritis (n = 60), psychiatric disorder (n = 86), diabetes (n = 54), glaucoma (n = 49), back disease (n = 367), arthritis (n = 244), empyema (n = 106), other chronic disease(s) except chronic bronchitis/bronchiectasis (n = 449). Education level = baccalaureate/secondary versus primary school; professional training = completed professional college/university/courses/trade school versus no professional training. OR = odds ratio. CI = confidence interval. Severe asthma (Sev-OCS) was defined as those who reported regular use of oral corticosteroid in e and/or ≥ 2 corticosteroid courses/year due to asthma

## Discussion

In this population-based case–control study of adult-onset asthma, severe asthma was associated with male sex, smoking, NERD, comorbidities, and the number of siblings. Notably, associations for smoking, NERD and comorbidity were found in a sensitivity analysis based on another asthma severity definition, indicating that these associations were robust to the asthma severity definition. The effects of these risk factors seem to be cumulative; each additional risk factor gradually increases the risk of severe asthma.

In our data, the prevalence of severe asthma was 7.4% in patients with adult-onset asthma. This finding is in line with previous observations, in which the prevalence of severe asthma has been estimated to vary between 5 and 10% of patients with asthma [8]. A Finnish single-centre (central hospital) cohort of unselected patients with adult-onset asthma estimated that 5.9% fulfilled the ERS/ATS criteria for severe asthma [9]. This lower proportion could be explained by different definitions of severe asthma than in our study.

Our results reinforced previous findings regarding the impact of NERD, smoking and comorbidities in severe adult-onset asthma. A systematic review identified 27 publications, in which the prevalence of NERD among asthmatic patients was approximately 7%, and it was the highest among patients with severe asthma [17]. An analysis of the Korea Severe Asthma Registry (n = 489) showed that individuals with severe asthma (including early- and adult-onset) have comorbidities such as allergic rhinitis (59%), atopy (39%), and aspirin hypersensitivity (14%) [40]. Severe asthma and/or NERD has been shown to be associated with type 2 inflammation [11]. Our findings could support that NERD, often characterized by type 2 inflammation in the literature [41], is an important risk factor for severe adult-onset asthma. Consistent with the literature, smoking and comorbidities were important independent factors of severe adult-onset asthma in our study. The deleterious effect of smoking in subjects with asthma has been well demonstrated in the literature, with decreased lung function [37], increased asthma severity [42], and a risk of mortality [8]. In a cohort of Finnish middle-age asthmatic patients (including early- and late-onset individuals) (n = 529), it was shown that 8% of asthmatic patients with more severe asthma and comorbidities had poorer Work Ability Scores during the 10-year follow-up [1].

Our study identified that independent of age and other factors, the presence of ≥ 2 siblings was a risk factor for severe adult-onset asthma. Professional training was associated with a lower risk of severe asthma, although the association was borderline significant in the multiple logistic regression. In our study population (composed of individuals born between 1904 and 1966), it could be speculated that the presence of ≥ 2 siblings could reflect poorer early living conditions predisposing patients to lower SES in adulthood [43], which thus may have an impact on asthma self-care behaviour [44]. A study of elderly French women (n = 2258) showed that a low educational level (11%) was associated with an increased risk of uncontrolled asthma (including early- and late-onset cases) [15]. In agreement with the hygiene hypothesis, a number of siblings have been suspected to protect against the development of childhood asthma [45] and other atopic diseases [46]. On the other hand, the number of siblings might be a risk factor for asthma and poor lower lung function because it might lead to increased contact with pathogens that cause lower respiratory infections [47] and may lead to exacerbated asthma, especially in genetically predisposed individuals [48].

Regarding sex, the main analysis showed a higher risk of severe adult-onset asthma in men than in women, but the sensitivity analysis using the OCS-based definition did not. This could reflect that male asthmatic patients reporting more difficult symptoms could have OCS-resistant asthma, such as smoking-related inflammation [49].

Our study did not detect an association between a self-reported history of severe childhood infection(s) and severe adult-onset asthma, which might be because childhood infections increase the risk of exacerbated childhood-onset asthma [48] more than severe adult-onset asthma. Another possible explanation might be related to measurement error/cohort effects, as since the decades when our study population was born, the treatment of childhood infections has changed due to the increased availability of public children’s counselling, doctors and antibiotics. There is no or little previous evidence of an effect of childhood infections on severe adult-onset asthma. In the Tasmanian Longitudinal Health Study (n = 7312), a history of pneumonia before the age of 7 years was ascertained from parents and measles, rubella, mumps, chickenpox, diphtheria, and pertussis from school medical records [36]. Greater infectious disease load was negatively associated with persisting asthma at all ages [36].

The presence of allergic disease(s) (AR and/or AC and/or AD) was not associated with the risk of severe adult-onset asthma in our study. In terms of mortality among asthmatic patients and matched controls, our previous study showed that the presence of AR or AC did not explain excess mortality among asthmatic adults [7], which is in line with our current findings. Overall, it is likely that asthma with allergic multimorbidity represents a phenotype that considerably differs from asthma alone in terms of mechanisms, severity and prognosis. Further studies in younger populations are needed, as we demonstrated earlier that the association between allergic multimorbidity and asthma differs with age, with a stronger association observed in the youngest population [24].

Although there is growing evidence that early-life factors play a role in the development of asthma (i.e., parental smoking, infection, nutrition, rural environment) [50], whether these early-life factors are associated with severe adult-onset asthma remains to be addressed. In our study, growing up in the countryside or parental asthma/allergy/smoking were not associated with severe adult-onset asthma in the main analysis, but the sensitivity analysis resting on the OCS-based definition of severe asthma showed a significant association with growing up in the countryside/on a farm. This could reflect OCS-sensitive inflammation (such as allergic fungal asthma). Other studies have shown that farm environments represent a source of fungi and increase asthma risk and that sensitization to fungi might be related to severe asthma [20], and the severity of allergic fungal asthma can be decreased by OCS [51]. Our previous analysis discovered an association between sensitization to *Aspergillus fumigatus* and asthma in an adult population [52].

Previous studies have shown that obesity increases the odds of a more persistent and severe asthma phenotype  [53] and that obesity-associated severe asthma may represent a distinct clinical phenotype [54]. However, we did not detect an association between BMI and severe adult-onset asthma in the main analysis, but a trend for a positive association was observed when using the medication-based definition of severe asthma.

This study has several strengths, including the outcome definition. In this population-based study, a specific definition of asthma was used based on lung-function-confirmed doctor-diagnosed asthma. Asthma is a clinical syndrome of chronic airway inflammation with variable bronchial obstruction and a heterogeneous background. Many epidemiological studies aiming at identifying risk factors for asthma have not considered this heterogeneity in disease expression, which may affect the interpretation and comparison of results between studies. In this study, we looked for risk factors for severe adult-onset asthma, a specific phenotype associated with a poor prognosis, with a definition of severity combining several domains of the disease. A further strength relies on the analytical approach, consisting of estimating the joint effect of multiple risk factors and addressing the robustness of the results to the definition of severe asthma. We chose two severe asthma definitions based on data available to test the robustness of our findings to the definition of severe asthma. Although only 41% of patients had severe asthma by both the Sev-Q and Sev-OCS definitions, these two severe asthma definitions showed consistency for three risk factors (smoking, comorbidities and NERD), supporting the role of these factors in severe asthma.

The weaknesses of our study include the limited statistical power of multivariable analyses. Due to the cross-sectional design of our study, we were not able to evaluate the causal direction of associations; however, because asthma was recently diagnosed (past year) according to the study design, covariates should have occurred before the development of adult-onset asthma. We acknowledge that we lacked lung function test data as an additional objective measurement of severe asthma and that a small portion of asthmatic patients might have childhood-onset asthma that relapses in adulthood. In addition, a memory bias in the reporting of risk factors might have occurred. We acknowledge that the data source was from 1997; therefore, the results should be applied to the present time with caution. We acknowledge that using a stringent definition of severe asthma still leaves the possibility that in some patients, asthma control could have been achieved by adequate doses of ICS or other inhaled medications instead of OCS. This was not possible to analyse, as detailed data on medication doses and indications were not available. The underuse of ICS was common in Finland in the early 1990s. After the launch of the Finnish asthma programme, the use of ICS and other inhalers rapidly improved, as has been shown by reduced asthma mortality and hospitalization days and an improved ratio between daily doses of preventers and relievers, which was 1.5 in 1996 [54]. Finally, the associations reported could be partly biased by residual confounders, either due to missing potential confounders in the regression model (such as occupational exposure) or due to limited accuracy in the assessment of some independent variables (i.e., the smoking variable does not consider the amount and duration of smoking). Thus, before extrapolating our results, replication studies are needed.

## Conclusion

Our study indicates that male sex, smoking, NERD, comorbidities, and ≥ 2 siblings are independent risk factors for self-reported severe adult-onset asthma. Although these results need validation in other populations, in terms of clinical implications, they reinforce the need for smoking cessation and the importance of diagnostics and the management of NERD and other comorbidities to prevent severe asthma in adult-onset asthma patients.

## Data Availability

The datasets generated and analysed during the current study are not publicly available because they contain information that could compromise research participant privacy but are available from the corresponding author upon reasonable request. A web link to data is not available.

## Abbreviations

AC: Allergic conjunctivitis
AD: Allergic dermatitis
AR: Allergic rhinitis
CI: Confidence interval
FEV1: Forced expiratory volume during the first second
ICS: Inhaled corticosteroid(s)
NERD: Non-steroidal anti-inflammatory drug (NSAID)-exacerbated respiratory disease
OCS: Oral corticosteroid(s)
OR: Odds ratio
PEF: Peak expiratory flow
SABA: Short-acting beta agonist(s)
SII: Social Insurance Institution of Finland
